# Patent foramen ovale diagnosis in young stroke patients: analysis of recurrence and mortality

**DOI:** 10.1007/s00415-025-13178-x

**Published:** 2025-06-11

**Authors:** Eva Giralt-Steinhauer, Elisa Cuadrado-Godia, Ana Rodriguez-Campello, Isabel Fernández-Pérez, Daniel Guisado-Alonso, Adrià Macias-Gómez, Marta Vallverdú-Prats, Julia Peris-Subiza, Sergio Vidal-Notari, Mireia Ble-Gimeno, Jordi Jiménez-Conde, Angel Ois, Joan Jiménez-Balado

**Affiliations:** 1 Neurology Department, Hospital del Mar Barcelona, Passeig Marítim 25-28, 08003 Barcelona, Spain; 2 Neurology Department, Hospital del Mar Barcelona, CEXS-Universitat Pompeu Fabra, Universitat Autònoma de Barcelona, Barcelona, Spain; 3 Hospital del Mar Research Institute Barcelona, Barcelona, Spain; 4 Cardiology Department, Hospital del Mar Barcelona, Barcelona, Spain

**Keywords:** Patent foramen ovale, Ischemic stroke, Guidelines, Incidence, Recurrence, Mortality

## Abstract

**Background:**

Revised European Stroke Organization guidelines in 2018 recommend early patent foramen ovale (PFO) detection and closure in patients aged 60 or younger who suffered an ischemic stroke. Our primary aim was to analyze the impact of these guidelines on the detection of PFO. Our secondary endpoints were to investigate the differences in the risk of recurrence and mortality among PFO status.

**Methods:**

We conducted a population-based, retrospective cohort study in Catalonia using linked health administration databases. We included all ischemic stroke patients aged 18–60 from 2016 to 2021, collecting PFO diagnosis, demographics, comorbidities, stroke recurrence, and mortality.

**Results:**

A total of 13,780 individuals suffered an ischemic stroke, representing a raw annual incidence rate of 30.3 cases-per-100,000 inhabitants/year. PFO was detected in 749(5.4%), and these were younger, and had a lower prevalence of risk factors than patients without PFO (all *p* value < 0.05). After adjusting for age and sex, PFO diagnoses increased by 59% following the guidelines update. Five-year recurrence was 12.1% [95%CI 11.3–12.9] with no differences by age and PFO. Socioeconomical status and diabetes emerged as predictors of recurrence. Stroke patients with PFO showed a lower mortality rate (*p* value = 0.016). However, when stratified by age, PFO was linked to lower 4-year mortality only in patients ≤ 50 years.

**Conclusions:**

We confirm a greater detection of PFO in real-world practice following the update of guidelines. Regarding the risk of recurrence, socioeconomic status and diabetes were the only independent predictors of new stroke events. Additionally, we found a lower all-cause mortality in younger patients with PFO.

**Supplementary Information:**

The online version contains supplementary material available at 10.1007/s00415-025-13178-x.

## Introduction

Ischemic stroke in young adults is a serious event that can cause death, lifelong disability, and decreased quality of life, with major social and economic impacts because of high healthcare costs. Worldwide, more than two million young adults suffer from ischemic stroke every year [1, 2]. Recent studies have revealed wide variation in the geographic incidence [3–5]. Young stroke patients are typically considered to be individuals who experience a stroke before the age of 50. However, the age cutoff is biologically arbitrary, and other definitions may extend the upper age limit to 60 [6]. Despite the ever-increasing number of ischemic strokes among younger patients [7], the causes of stroke remain unknown in about one-third of all patients. In these patients, the presence of a patent foramen ovale (PFO) may be a potential cause of paradoxical embolism. PFO exists in approximately 25% of the population, with high-risk features likely associated with clinical events in presumably 5% [8]. The clinical benefit of PFO closure was previously uncertain. However, several trials have provided data on the role of PFO closure for secondary stroke prevention [9–12]. Consequently, the European Stroke Organization (ESO) state in 2018 that it is reasonable to recommend PFO closure in patients aged between 18 and 60 with embolic appearing stroke, with no other apparent mechanism [13], introducing an alternative therapeutic option that can significantly change patient management.

Despite the publication of clinical guidelines, there is a relative scarcity of studies evaluating their impact on real-world clinical practice. This gap in the literature is particularly evident in the context of PFO diagnosis, where evolving guidelines have the potential to significantly influence clinical decisions.

Conversely, stroke outcome is particularly important for young patients due to their extended life expectancy and significant responsibilities during a crucial phase of life. Information regarding specific subgroups of younger ischemic stroke patients with respect either to stroke recurrence or mortality, is scarce and primarily based on smaller studies [14]. These studies indicate that younger ischemic stroke patients have a similar rate of stroke recurrence compared to older individuals and also face an increased long-term mortality relative to the general population [15, 16].

Therefore, our hypothesis is that revised European guidelines for the diagnosis and treatment of PFO in 2018 has resulted in greater detection of PFO in patients who might have previously gone unnoticed. This study aims to describe the number of strokes in young adults in Catalonia between 2016 and 2021 and to determine if the changes in the European guidelines increases the diagnosis, across the Catalan territory, of PFO in stroke patients. Our secondary objective is to analyze the differences between those with PFO diagnosis and those without, regarding comorbidities, risk of recurrence after the initial stroke, and mortality, filling an important gap in the current literature.

## Methods

### Data sources and study population

We conducted a population-based, retrospective cohort study. Anonymized and unidentified data were extracted by Data Analytics Program for Health Research and Innovation (PADRIS) of the Agency for Health Quality and Assessment of Catalonia (AQuAS). The database contained data from different healthcare providers, which were updated annually during the study period (from 2016 to 2021) and, in some cases, for several years before (see Supplementary Fig. 1). Specifically, we considered data from primary care, hospitalizations and emergency care. These databases aggregate data from patients’ diagnoses, such that one row represents an individual patient diagnosis. To link these datasets, we used a unique random identification hash code assigned to each patient [17]. Patient diagnoses were recorded using the ninth or tenth revisions of the International Classification of Diseases codes (ICD-9 and ICD-10).

For our study, we identified patients aged 18–60 who experienced an ischemic stroke between 2016 and 2021 (ICD9: 433, 434, 435, and subcodes, except 435.2; ICD10: I63.xx except I63.6; emergency room and hospitalization databases; Supplementary Table 1). This yielded a cohort of 13,780 individuals (Supplementary Fig. 2). In cases where a patient had multiple ischemic events during this period, we designated the initial occurrence as the index stroke. The primary outcome was to determine if the update in the European guidelines increases the diagnosis of PFO in stroke. So, we then searched for each patient entries indicating a diagnosis of PFO (ICD9: 745.5; ICD10: Q21.1), retaining the first instance in cases where multiple entries were found. For patients diagnosed with PFO, we calculated the time from index stroke to PFO diagnosis.

### Variables and outcomes

#### Demographic data

Patient-level demographic variables, as registered in the primary healthcare database, included age at the index stroke, sex at birth, territory and socioeconomic status. Age was categorized into 4 groups: 18–29, 30–39, 40–49, and 50–60 years. We also examined potential territorial disparities in PFO diagnosis. Catalonia has 8,016,606 inhabitants, according to the January 2024 population census and is partitioned into several healthcare regions, known as"Regions Sanitàries"—sanitary regions— each responsible for administering and delivering healthcare services within its designated area. These regions encompass Barcelona (“Barcelona Ciutat”), North Barcelona (“Barcelona Metropolitana Nord”), South Barcelona (“Barcelona Metropolitana Sud”), Penedès, Terres de l'Ebre, Central Catalonia (“Catalunya Central”), Camp de Tarragona, Girona, Lleida, and Alt Pirineu i Aran. Information regarding individual socioeconomic status was inferred from the percentage of pharmacy co-pay, which depends on the patient’s annual income. Annual income was categorized as follows: annual income below 18,000€; annual income from 18,000 to 100,000€; and annual income exceeding 100,000€.

#### Vascular risk factors and comorbidities

Regarding history of vascular risk factors, we gathered information on hypertension, diabetes, dyslipidemia, atrial fibrillation (AF), and obesity. To achieve this, we screened records mainly from primary care dating back to the year 2000 (Supplementary Fig. 1). We identified relevant entries labeled for any of these risk factors according to the ICD-9 and/or ICD-10 (Supplementary Table 1). A history of any of these vascular risk factors was defined by the presence of an entry recorded before the date of the index stroke. For history of previous comorbidities, we followed the same procedure and recovered data about previous ischemic stroke, ischemic heart disease, thrombophilia, venous thrombosis and migraine (Supplementary Table 1).

#### Antiplatelet and anticoagulant treatments

To assess antiplatelet and anticoagulant treatments, we reviewed pharmacy records containing prescription data. We filtered entries based on relevant ATC codes: anticoagulants (B01 AA*—vitamin K antagonists; B01 AE*—thrombin inhibitors; B01 AF*—factor Xa inhibitors) and antiplatelets (B01 AC*). For each patient, we identified the earliest recorded prescription for each drug category. We then compared the prescription date with the index stroke date to determine whether the treatment was initiated before or after the stroke event.

#### Stroke recurrence and mortality

Our secondary endpoints were stroke recurrence and mortality. Stroke recurrence was defined as a new ischemic stroke event at least 15 days after the index stroke date. We employed the same diagnostic codes as outlined in Supplementary Table 1. We specifically accounted for entries beyond the initial 15-day period following the index stroke to mitigate the possibility of duplicated entries and stroke diagnoses. Moreover, we only included patients without a previous history of stroke, in which the index stroke was the first ischemic stroke event. Finally, regarding patients without incident events, we only included those with at least a 3-month follow-up, resulting in a cohort of 9,180 individuals (Supplementary Fig. 2).

In terms of all-cause mortality, the dataset capturing mortality causes was only updated until 31/12/2020 (Supplementary Fig. 1). Consequently, we excluded stroke cases occurring in 2021 from our mortality analysis. The event of interest was all-cause mortality among 30-day stroke survivors [16]. For those who did not experience a fatal event, we further excluded patients with follow-up periods shorter than three months, yielding a sample of 9070 subjects to study the long-term survival (Supplementary Fig. 2).

For both secondary endpoints, we needed to define the last valid follow-up date for each patient. To achieve this, we merged entries from all databases (primary healthcare, pharmacy, emergency room, and hospitalizations) and recorded the date of the last entry.

### Ethical

Data from different health administration databases were linked and deidentified by a team not involved in the study analysis; only a full deanonymized database was available to the study investigators (J. Jiménez-Balado and E. Giralt-Steinhauer). The study protocol was approved by the Ethical Review Board of the Hospital del Mar Barcelona (2022/10458), Barcelona, Spain. The STROBE guidelines were used to ensure the reporting of this observational study.

### Statistical analysis

All analyses were performed using R statistical software, version R-4.0.

#### Descriptive analyses

Data were presented as mean (± standard deviation), median (interquartile range) or frequency (percentage) depending on the distribution of each variable. Demographic and clinical variables were compared between groups (PFO and no-PFO) using *χ*^2^-, *t*-, or *U* Mann–Whitney tests, as appropriate. Besides, we conducted sensitivity analyses excluding participants older than 50, when necessary.

The raw and age-specific annual stroke incidence was computed by dividing the number of stroke events occurring each year by the population of Catalonia for that specific year (comprising the entire population or those aged 18 to 60, respectively). These rates were expressed per 100,000 inhabitants. Additionally, we examined the incidence stratified by age, specifically comparing patients older and younger than 50 years, as well as by decade of life. To evaluate temporal changes in stroke incidence, Kendall’s rank correlation tests were utilized. For each incidence rate, we calculated the 95% confidence intervals as described elsewhere [18].

#### PFO diagnoses

To evaluate the rise in PFO detection during the study period, we conducted Cochran–Armitage tests at the univariate level. Furthermore, we compared PFO detection before (2016–2017) and after (2019–2021) the update in clinical guidelines (2018) using a *χ*^2^ test. Confidence intervals for proportions were calculated using a method described by Wilson et al. [19].

We continued doing multivariate analyses in which the presence or absence of PFO was considered as a binomial variable. Hence, we employed multivariate logistic regression models with PFO diagnosis as the dependent variable at the patient-level and dichotomous time (2016–2017 vs 2019–2021) as the independent predictor of interest. This model was adjusted for continuous age to address demographic differences across periods. Besides, we conducted an additional model considering the entire study period from 2016 to 2021, where the year was introduced as a continuous variable in exchange of dichotomic time. This model tested whether existed a linear increase in PFO diagnosis over the full follow-up. Finally, to explore potential non-linear relationships, we incorporated year as an ordinal variable and employed backward difference contrasts. This method enabled us to compare the average proportion of diagnosed PFO between consecutive years and assess any deviations from linear trends. These analyses were stratified by the sanitary region although we excluded the Pirineu region due to its small population size and low stroke incidence and, thus, we could not calculate confidence interval for this territory.

We also tested whether the effect of time, either as a dichotomic, continuous or ordinal variable, on PFO diagnosis was influenced by relevant demographic variables, such as age, sex and sanitary region. To that aim, we modeled the interaction between time and these predictors.

#### Stroke recurrence

To assess the determinants of stroke recurrence in the sample, we focused exclusively on subjects with valid follow-up data and no previous history of ischemic stroke, extending at least 3 months after stroke onset (*N* = 9180). Additionally, to manage censored cases effectively, we right-censored the follow-up period at 5 years. To compare the incidence of new stroke events between participants with and without PFO, we employed Kaplan–Meier curves and conducted log-rank tests. We also conducted additional series of Kaplan–Meier analyses to report which clinical and demographic variables were associated with stroke recurrence at the univariate level.

We subsequently constructed multivariate Cox regression models employing a forward stepwise algorithm based on the Bayesian Information Criterion (BIC) to select variables. Covariates included in the fully adjusted model were those that exhibited associations with stroke recurrence at the univariate level. Therefore, we adjusted for age, sex, vascular risk factors, and previous ischemic heart disease. After obtaining the most informative set of covariables, we entered PFO into this baseline model, allowing us to understand the effect of PFO on the incidence of recurrent stroke independently of potential confounders.

#### All-cause mortality

All-cause mortality in 30-day stroke survivors was reported in 9070 patients with valid follow-up (see the previous section). Additionally, we right-censored the follow-up period at 4 years to mitigate the potential for excessive dropouts during follow-up, as we did for stroke recurrence. The yearly survival rate was determined using the Kaplan–Meier estimator. To compare mortality rates between groups, log-rank tests were employed.

## Results

### Characteristics of the cohort

**Fig. 1:**
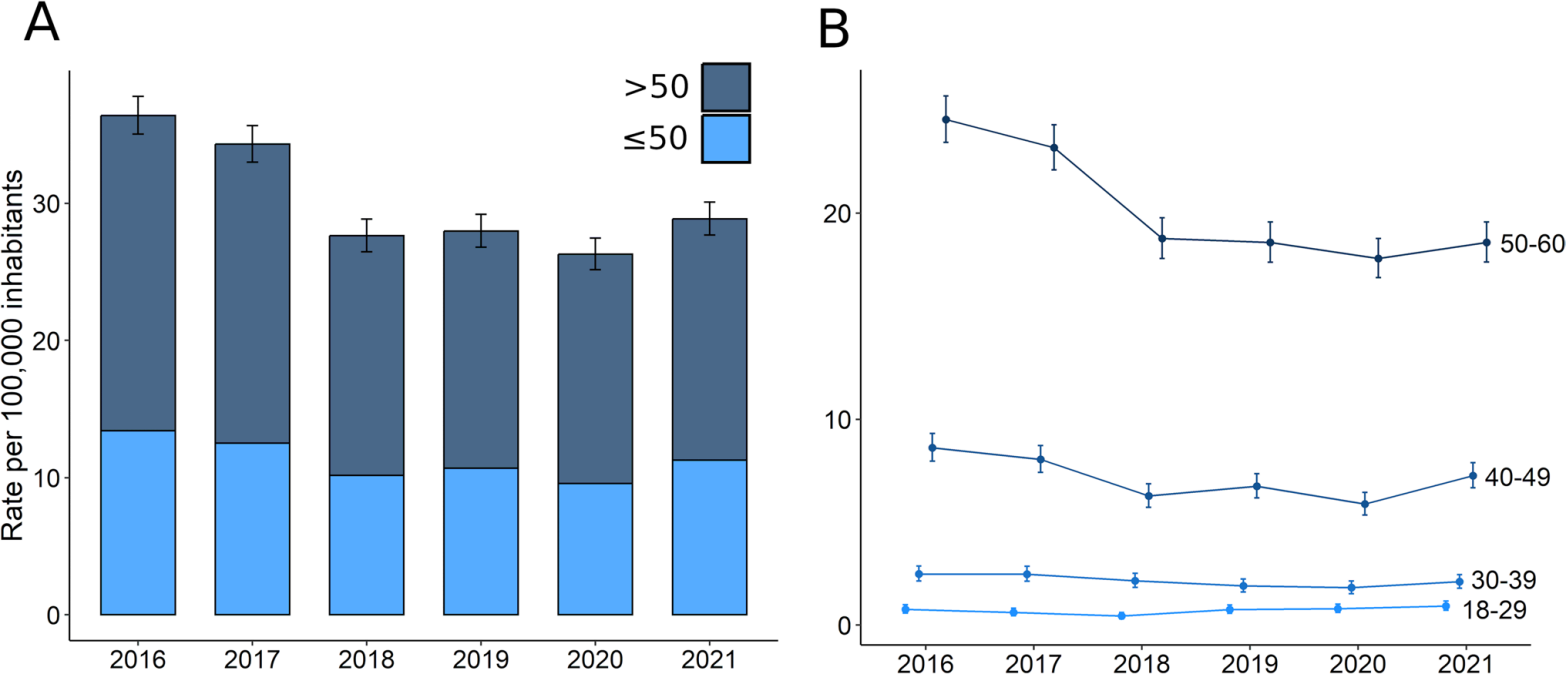
Incidence of stroke at younger ages. Panel **A** depicts the raw incidence rates per 100,000 inhabitants by age (individuals older and younger than 50). Panel **B** stratifies the incidence rates by decades of life

A total of 13,780 individuals aged between 18 and 60 years suffered an ischemic stroke from 2016 to 2021 in Catalonia, representing a global raw rate of 30.3 cases per 100,000 inhabitants-year (95% CI 29.8–30.9). As expected, individuals aged 18–50 had a lower incidence (11.3 cases per 100,000 inhabitants-year, 95% CI 11.0–11.6) compared to those older than 50 (19.0 cases per 100,000 inhabitants-year, 95% CI 18.6–19.4). We observed no change in the rate of events within time in the whole sample (*τ* = − 0.47, *p* value = 0.272; Fig. 1A) although we observed a trend toward a slight decrease in participants aged between 50 and 60 (*τ* = − 0.73, *p* value = 0.056; Fig. 1B). Similar trends were observed for age-specific incidence rates as showed in Supplementary Fig. 3.

General characteristics of the sample are summarized in Table 1. PFO was detected in 749 (5.4%) individuals, and most of them (71.3%) were diagnosed within the first month after index stroke (Supplementary Fig. 4).

**Table 1:** Main characteristics in the cohort and by presence of PFO

	Whole cohort	Stroke patients with PFO	Stroke patients without PFO	*p* value
	*N* = 13,780	*N* = 749	*N* = 13,031	
Demographics				
Age, years	53 (47–57)	49 (42–55)	53 (47–57)	< 0.001
Age in decades				< 0.001
18–29	326 (2.4)	42 (5.6)	284 (2.2)	
30–39	984 (7.1)	106 (14.2)	878 (6.7)	
40–49	3257 (23.6)	245 (32.7)	3012 (23.1)	
50–60	9213 (66.9)	356 (47.5)	8857 (68.0)	
Age ≤ 50 years	5143 (37.3)	436 (58.2)	4707 (36.1)	< 0.001
Sex, female	4597 (33.4)	289 (38.6)	4308 (33.1)	0.002
Socioeconomic status				< 0.001
< 18,000€	10,187 (76.5)	482 (65.9)	9705 (77.1)	
18,000–100,000€	3067 (23.0)	245 (33.5)	2822 (22.4)	
> 100,000€	59 (0.52)	4 (0.55)	65 (0.52)	
Vascular risk factors				
Hypertension	5268 (38.2)	135 (18.0)	5133 (39.4)	< 0.001
Diabetes	2615 (19.0)	57 (7.6)	2558 (19.6)	< 0.001
Dyslipidemia	4418 (32.1)	161 (21.5)	4257 (32.7)	< 0.001
Atrial fibrillation	927 (6.7)	26 (3.5)	901 (6.9)	< 0.001
Obesity	3853 (28.0)	165 (22.0)	3688 (28.3)	< 0.001
Previous comorbidities				
Stroke	1789 (13.0)	99 (13.2)	1690 (13.0)	0.888
Ischemic heart disease	880 (6.4)	21 (2.8)	859 (6.59)	< 0.001
Thrombophilia	69 (0.5)	3 (0.4)	66 (0.5)	1.000
Venous thrombosis	81 (0.6)	3 (0.4)	78 (0.6)	0.803
Migraine	971 (7.1)	75 (10)	896 (6.9)	0.001
Previous use of antiplatelets and oral anticoagulants drugs				
Antiplatelets	2591 (18.8)	93 (12.4)	2498 (19.2)	< 0.001
Anticoagulant	480 (3.5)	20 (2.7)	460 (3.5)	0.252
New use of antiplatelets and oral anticoagulants drugs				
Antiplatelets	7581 (55.0)	525 (70.1)	7056 (54.1)	< 0.001
Anticoagulant	883 (6.4)	102 (13.6)	781 (6.0)	< 0.001

Values represent frequencies (percentages) or medians (interquartile ranges). Characteristics between participants with and without PFO have been obtained with *χ*^2^ or *U* Mann–Whitney tests, accordingly

If we consider only patients aged ≤ 50 years (*N* = 5143), there were 436 (8.5%) cases with PFO. In general terms, stroke patients with PFO tended to be younger and more frequently female, with a significantly lower prevalence of all vascular risk factors and comorbidities, except for migraine, which exhibited a higher frequency in stroke patients with PFO (Table 1). Moreover, patients without a PFO had a higher rate of antiplatelet use prior to the stroke, whereas those with a PFO were more frequently prescribed antiplatelet or anticoagulant therapy following the stroke. When we considered only patients aged 50 or younger, we observed similar differences between patients with and without PFO, with the exception of sex at birth and atrial fibrillation variables, which showed no significant differences between groups in this subset of the cohort (Supplementary Table 2).

### PFO diagnosis

**Fig. 2:**
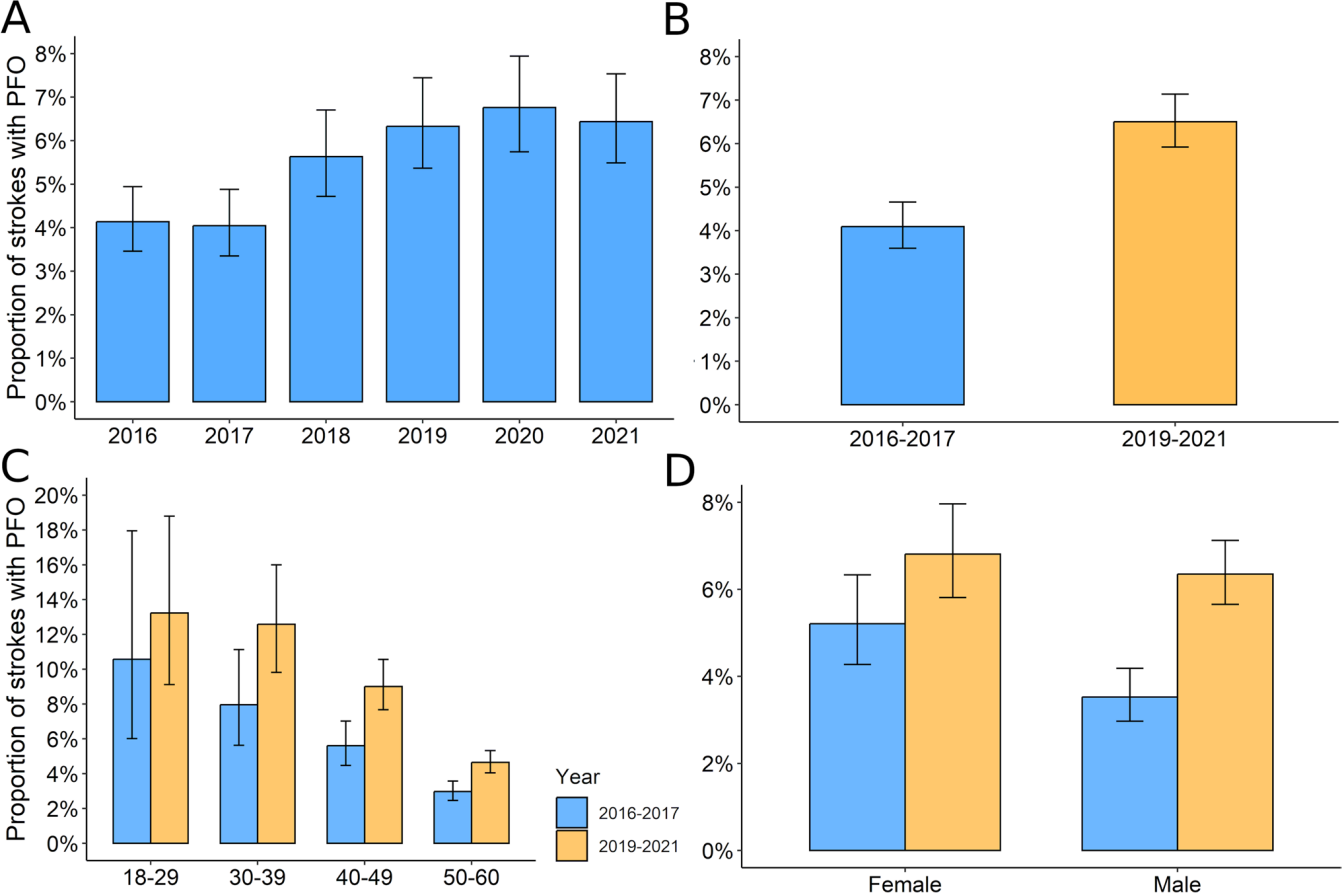
Change in the trend of PFO diagnosis within time. Panel **A** displays de increase in PFO diagnosis within the full study period. Panel **B** compares this increase in the periods before (2016–2017, blue) and after (2019–2021, orange) the update in clinical guidelines. We additionally show how this trend differs according to age of participants (panel **C**) and sex (panel **D**)

We identified a statistically significant temporal trend in the rise of PFO diagnoses (Cochran–Armitage test, *χ*^2^ = 28.02, *p* value < 0.001; Fig. 2A). When we compared the period before and after the update of clinical guidelines (2016–2017 vs 2019–2021), we confirmed an increase in the percentage of stroke cases diagnosed with PFO (PFO_2016-2017_ = 4.1% vs PFO_2019-2021_ = 6.5%; *χ*^2^ = 32.50, *p* value < 0.001; Fig. 2B). Importantly, in Supplementary Table 3, we compare subjects without PFO screened at both periods, where we show that there were no differences in critical variables, such as continuous age and sex between groups. We only found that subjects who had the stroke between 2019 to 2021 had a lower prevalence of diabetes, dyslipidemia, previous stroke and ischemic heart disease, while a higher prevalence of obesity (all *p* value < 0.05). Similarly, there was a slightly higher prevalence of subjects aged 18–29 in the period from 2019 to 2021 (1.94% vs. 2.98%; see Supplementary Table 3). We also compared the characteristics of patients with PFO recruited during both periods and observed no differences in demographic variables, vascular risk factors, or comorbidities (Supplementary Table 4).

After adjusting for age and sex, our analysis revealed a 59% increase in the proportion of strokes with PFO following the update in clinical guidelines (OR 1.59 [95% CI 1.35 to 1.89], *p* value < 0.001; Fig. 2B and Supplementary Table 5). Remarkably, although we observed a linear trend in the rise of PFO detection within this cohort, this increase plateaued in 2018–2019 and remained stable until the conclusion of the follow-up period (Fig. 2A and supplementary Table 5).

We also examined whether the increase in PFO diagnoses was consistent across all age groups and sex. After studying the interaction term between time and age group, we confirmed there was no moderation of age on the relationship between time and PFO diagnosis (OR 1.01 [95% CI 0.99–1.02], *p* value = 0.416; Fig. 2C and Supplementary Tables 6 and 7). Although no significant interaction between sex and categorical time was observed, the increase in PFO detection following the update in clinical guidelines was higher in men (OR 1.38 [95% CI 0.98–1.96], *p* value = 0.070; Fig. 2D and Supplementary Tables 6 and 7).

### Regional distribution

We proceeded to evaluate the impact of the updated clinical guidelines on each sanitary region in Catalonia. Most regions showed an increase in the detection of PFO following the update of clinical guidelines, with only Lleida and Penedès locations showing stability or decrease (supplementary Fig. 5-A). Although the interaction between region and time did not yield statistical significance (*F* = 0.57, *Df* = 9; *p* value = 0.825; Supplementary Tables 6 and 7), we visually depicted the linear relationship between time and prevalence of PFO diagnosis on the map of Catalonia, reaffirming a widespread increase in PFO detection across most Sanitary regions over time (supplementary Fig. 5-B).

### Stroke recurrence

To study the incidence of recurrent stroke, we analyzed a subset of the cohort consisting of 9180 individuals without prior history of ischemic stroke and who had a valid follow-up within the 3 months after stroke onset. In the Supplementary Table 8, we compare the baseline characteristics of patients included in the sub-study with those who were excluded. Notably, the included patients were slightly older and exhibited a higher prevalence of risk factors compared to the excluded group. The median follow-up duration was 3.1 years (IQR 1.5–5.0 years), encompassing a total of 27,758.8 patient-years. Over this period, there were 917 (9.99%) stroke recurrences, representing a 5-year incidence of 12.1% [95% CI 11.3–12.9]; supplementary Fig. 6).

In the univariate analyses, we observed that the socioeconomic status was associated with 5-year stroke recurrence, such that subjects with a lower social status experienced a higher incidence of new strokes (*p* value < 0.001, Supplementary Table 9). Likewise, presence of diabetes and previous ischemic heart disease were all linked to stroke recurrence at 5 years in the univariate analyses (*p* value < 0.05). However, we found no differences in stroke recurrence between cases with and without PFO (Incidence_PFO_: 12.9% [95% CI = 9.7–16.2] vs Incidence_No-PFO_: 12.1% [95% CI = 11.3–12.9], *p* value = 0.830; Supplementary Table 9).

We continued studying which of these variables were independently associated with multivariate cox models. As displayed in Table 2, when we analyzed the whole sample, we observed that socioeconomic status significantly predicted new stroke events. Specifically, patients with an annual income lower than 18,000€ had a 1.43-fold increased likelihood of events compared to those with an annual income higher than 18,000€. Additionally, diabetes was also independently associated with stroke recurrence (*p* value < 0.001, Table 2). After adjusting for these confounders, PFO was not related to stroke recurrence (HR 1.13 [95% CI = 0.87–1.47], *p* value = 0.369). When we stratified the cohort into participants with and without PFO, a similar predictive model emerged for those without PFO. However, in the subset of participants with PFO, only diabetes showed a marginal effect (*p* value = 0.06) on the risk of new stroke events, with no effect of socioeconomic status.

**Table 2:** Independent predictors of stroke recurrence

Variable	HR (95% CI)	*p* value
Whole sample		
Socioeconomic status		
≥ 18,000%	REF	REF
< 18,000%	1.43 (1.21; 1.70)	< 0.001
Diabetes	1.45 (1.25; 1.69)	< 0.001
Presence of PFO	1.13 (0.87; 1.47)	0.369
Patients with PFO		
Diabetes	2.03 (0.96; 4.27)	0.063
Patients without PFO		
Socioeconomic status		
≥ 18,000%	REF	REF
< 18,000%	1.47 (1.22; 1.76)	< 0.001
Diabetes	1.43 (1.23–1.67)	< 0.001

Values represent hazard ratios (HR) and 95% confidence intervals (CI). Models have been obtained by a forward selection algorithm based on Bayesian Information Criterion (BIC)

### All-cause mortality

**Fig. 3:**
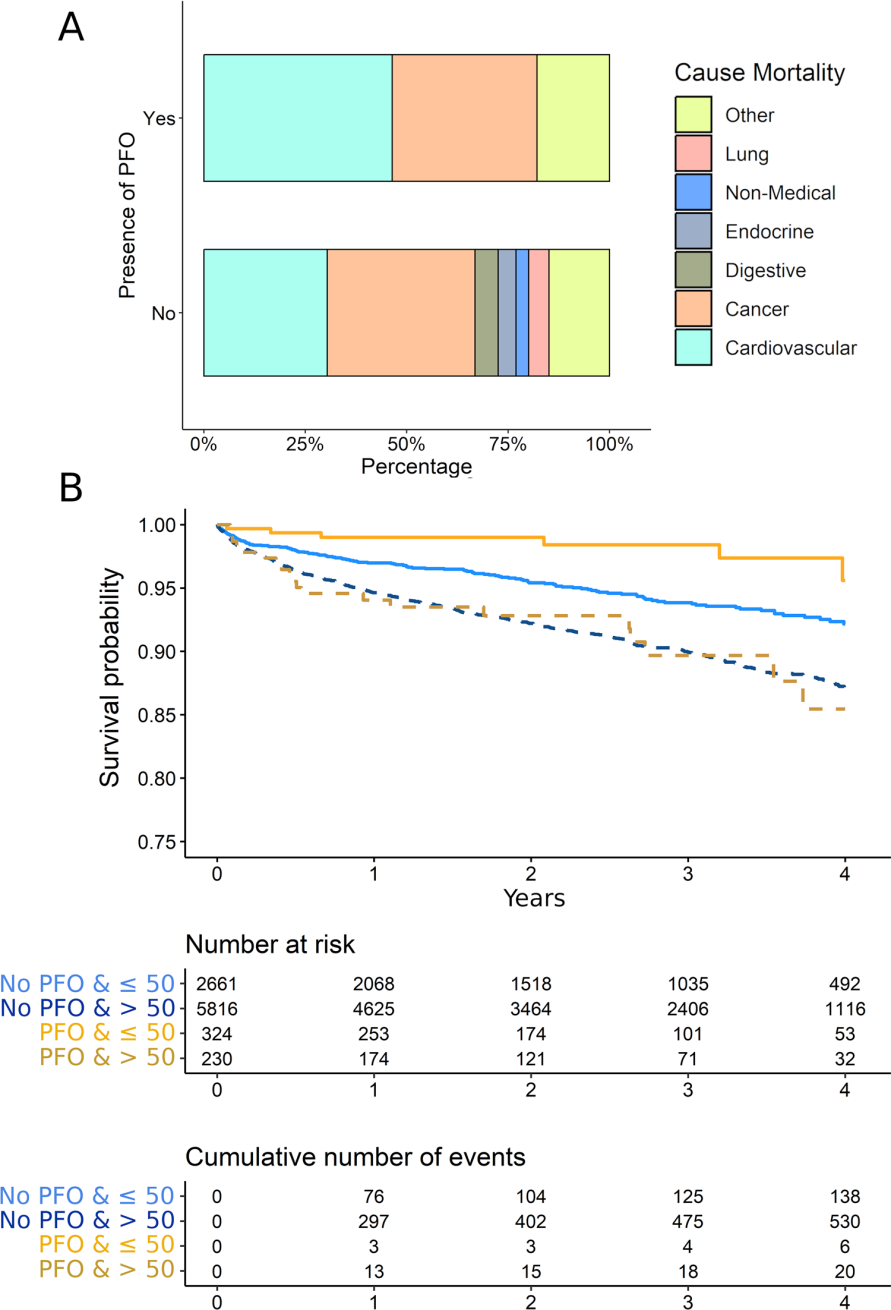
All-cause mortality in the cohort by presence of PFO. Panel **A** compares the causes of death between those patients with and without PFO. Panel **B** depicts the survival function in the sample by age (older or younger than 50) and by presence of PFO. Risk depicts the number of patients at risk and the cumulative number of events by groups

We studied mortality in 9070 patients who survived the index stroke, had a follow-up duration of at least 3 months (see the methods section). Among these patients, 705 (7.8%) died during the study period, with 217 (2.4%) of those deaths attributed to vascular causes. Rest of patients died from other causes including: cancer (*n* = 260, 2.9%), digestive (*n* = 38, 0.4%), endocrine (*n* = 30, 0.3%), lung (*n* = 34, 0.4%), non-medical (*n* = 22, 0.2%), and other causes (*n* = 104, 1.1%; blood, psychiatric, infectious and other neurological diseases a part from stroke). When we compared the causes of death between patients with and without PFO, we observed that those with PFO showed a higher percentage of cases dying due to vascular causes, while patients without PFO exhibited a wider variety of causes of death, although these differences were not statistically significant (*χ*^2^ = 12.09, *p* value = 0.098; Fig. 3A).

Our follow-up included 21,442.4 patients-years, such that the median follow-up duration was 2.5 years (IQR 1.2–3.7 years). During this period, the 4-year mortality rate was 11.3% [95% CI 10.4–12.1]. As expected, fatal events were associated with age, such that younger patients presented a lower 4-year mortality rate as presented in Supplementary Table 10 (*p* value < 0.001). Interestingly, we observed that patients with PFO showed a lower mortality rate as compared to patients without (Mortality_PFO_: 8.6 [95% CI 4.7–12.5] vs Mortality_No-PFO_: 11.4 [95% CI = 10.5–12.3]; *p* value = 0.016). However, when we stratified these results by age, we found that PFO was associated with a lower 4-year mortality rate only in patients aged 50 years or younger. In this subset, the mortality rate was 4.4% [95% CI 0.3–8.5] for those with PFO, compared to 7.8% [95% CI 6.4–9.2] for those without PFO (*p* value = 0.019). This effect was not observed in patients older than 50 years, where the 4-year mortality rate was 14.5% [95% CI 6.8–21.6] for those with PFO, compared to 13.0% [95% CI 11.8–14.1] for those without PFO (*p* value = 0.983; see Fig. 3B).

## Discussion

In this large population-based, retrospective cohort study, we found that 13,780 individuals aged 18–60 suffered an ischemic stroke from 2016 to 2021 in Catalonia, representing a global raw incidence of 30.3 cases per 100,000 persons-year. Our incidence is similar to other countries, such as Switzerland and Ireland, but comparing our results to other European countries, the rates vary significantly [20]. But it is well known, that the incidence of ischemic stroke among younger individuals shows significant variation across different regions globally, explained by various factors, including ethnicity, sex, prevalence of risk factors, lifestyle, socioeconomic status, and healthcare quality [5].

Stroke patients with a PFO diagnosis represented a 5.4% of the cohort and tended to be younger, more often female, exhibiting a notably lower prevalence of all vascular risk factors. When we compared the period preceding and succeeding the update of clinical guidelines [21], we verified, across all age groups, a rise in the proportion of stroke cases with PFO identified. Moreover, after adjustment for age, our analysis revealed a 59% increase in the percentage of PFO diagnosis in stroke patients. This result could be attributed to several factors. First, the update in clinical guidelines might have raised awareness among healthcare professionals regarding the association between PFO and stroke. This leads to more comprehensive screening for detecting PFO in stroke patients. Second, the updated guidelines in 2018 have shifted treatment recommendations, encouraging healthcare providers to actively diagnose PFO in these patients for optimal treatment. And third, new research findings or evidence emerging after the guidelines update may have highlighted the importance of PFO in stroke etiology, prompting increased attention and investigation into several specifics concerns [22].

In addition, our analysis indicates that the increase in PFO diagnoses was consistent across age groups, with no significant interaction between age and time, suggesting that the trend in increased diagnoses is not influenced by patient age. Interestingly, although there was no statistically significant interaction between sex and time, the data suggest a trend where the increase in PFO detection was more pronounced in men. Initially, PFO was diagnosed less frequently in men; however, clinical studies and meta-analyses, published between 2018 and 2019, have highlighted a potentially greater clinical benefit of PFO closure in male patients in the context of cryptogenic stroke [23–27]. This emerging evidence appears to have contributed to the observed increase in PFO diagnoses among men, reflecting a shift in clinical practice toward more frequent detection in this demographic [28].

Another interesting finding of our study, is the diagnostic heterogeneity in the different Sanitary Regions in Catalonia, most of them showing an increase in the detection of PFO, except for Lleida and Penedès. No previous data exist on the variation in PFO diagnosis within Catalonia, a region with a homogenous health system and universal insurance coverage. We hypothesized that perhaps one explanation to this variation is more likely due to differing practices of local physicians, which are usually more standardized in larger hospitals. Our research, alongside future epidemiological studies, will contribute to identifying trends across various regions. This insight will enable the implementation of targeted actions to enhance PFO detection.

In our study adults aged 18–60 years had a 5-year recurrence rate of 12.1%, with no effect of age on this risk. In a prior study, the cumulative 5-year recurrence rate after an ischemic stroke patients aged 15–49 years was 9.4% [95% CI 7.3–11.5] for nonfatal or fatal ischemic stroke [29]. Also, findings from a study that included 1216 patients aged 18–49 have reported a 5-year risk of any recurrent vascular event (defined as ischemic stroke, myocardial infarction, the revascularization procedures, or vascular death) of 12.2% [15]. Previous studies demonstrated that young ischemic stroke patients remain at significant risk of recurrent vascular events in the long term (10-year follow-up) as well [30, 31]. It is important to note that despite PFO patients have less vascular risk factors and comorbidities, we found no differences in stroke recurrence between cases with and without PFO, highlighting the need of an optimal secondary prevention tailored to their condition. To our knowledge, this is the first study among stroke patients to investigate if this difference exists. Our recurrence risk was higher as compared to a prior study based on the pooled control arms from 6 randomized clinical trials that compared PFO closure plus medical therapy vs medical therapy alone in patients with PFO-associated strokes [32]. Our observational and retrospective study may have included a more heterogeneous patient population, potentially with higher risk factors for stroke recurrence. The difference in study design can lead to variations in recurrence rates.

Moreover, our analyses showed that socioeconomic status significantly predicted new stroke events. Specifically, patients with a lower rent (< 18,0000€) had a 1.43-fold increased likelihood of events compared to those with a middle to high annual rent (≥ 18,000€) after adjusting for other confounders. The socioeconomic status is a recognized risk factor for stroke, influencing the likelihood of developing vascular risk factors, such as diabetes, high blood pressure, and smoking, and also appears to independently increase stroke incidence and mortality [33–36]. However, the effect of socioeconomic status on risk of recurrent stroke has been studied less extensively [34]. Three previous European studies reported no overall associations between income level, occupational class, or low SES and stroke recurrence [37–39]. But in a Swedish nationwide observational study they found that a lower educational level, low income, and living alone were all factors associated with an increased risk of stroke recurrence, also after adjusting for cardiovascular risk factors [40]. In a recent population-based study within the Greater Cincinnati/Northern Kentucky region, residents of poorer neighborhoods had a dose-dependent increase in stroke recurrence risk [41]. Despite Catalonia counts with a publicly financed healthcare system, we also observed social inequalities in stroke recurrence. We hypothesize that income may determine patients’ opportunities for healthy living and manage their disease accordingly. However, the underlying causes of this increased risk of recurrence among socioeconomically disadvantaged individuals in our region remain largely unknown. Additionally, diabetes was also independently associated with stroke recurrence. It is well known that diabetes significantly increases the risk of stroke, such that diabetic individuals are 2–5 times more likely to experience a stroke compared to those without diabetes, and they also exhibit a higher rate of recurrent strokes [42–45].

Among young and middle-aged adults, who were 30-day survivors of index stroke, the incidence of all-cause mortality after 4 years follow-up was 11.3% [95% CI 10.4–12.1], being cancer and cardiovascular events the most common causes of death. We found that patients with PFO showed a lower mortality rate as compared to patients without, but these differences were moderated by age, such that the incidence of all-cause mortality was only smaller in patients younger than 50. This suggests that younger patients with PFO may have better overall health or receive more effective treatments and follow-up care. On the contrary, patients older than 50 with PFO showed a mortality rate equivalent to those without PFO.

Our study has several strengths. Given the nearly universal public health coverage in Catalonia, it offers extensive data on the demographics, comorbidities, and socioeconomic status of nearly all stroke cases. All this information is recorded in a centralized program continuously monitored and updated, and has facilitated previous population studies utilizing a similar methodology [17]. To our knowledge, this is the first study to compare recurrence risk and mortality in such a large cohort.

This study has several limitations. First, the registry-based design may lead to an underestimation of PFO prevalence. Second, information on ischemic stroke subtypes and PFO characteristics was unavailable, preventing us from determining whether the diagnosed PFO is causal or incidental. Third, despite a major interest of this study was to determine if the updated guidelines have led to an increase in PFO closures, unfortunately, we were unable to report PFO closure rates, due to reliance on ICD coding, which we discovered not consistently performed. This information is crucial as our findings indicate that strokes with PFO have the same recurrence risk as those without PFO. However, it remains unclear whether the PFOs were closed, which could potentially influence the recurrence risk. Finally, patients who were excluded from the stroke recurrence sub-study due to lack of follow-up were slightly younger and had a lower prevalence of risk factors. Although these differences were small in size, they represent a limitation of our study. Further epidemiological research will be necessary to confirm the generalizability of our findings to other populations and settings.

## Conclusions

In conclusion, our results demonstrate that the diagnosis of PFO has increased in the younger stroke population following the 2018 guidelines update, independently of age and sex, across almost all healthcare regions in Catalonia. Notably, 1.2 in 10 stroke patients aged 18–60 experiences a recurrent stroke within 5 years, with no significant differences between patients with or without PFO. Similarly, this risk of recurrence was not affected by age, observing a similar rate of new stroke events across decades of life. Socioeconomic status and diabetes were as an independent predictor of recurrence. Stroke patients with PFO aged 50 years or younger exhibited a lower mortality rate compared to those without PFO. However, this difference in mortality was not observed in patients aged 51–60 years.

## Supplementary Information

Below is the link to the electronic supplementary material.

Supplementary file1 (DOCX 607 KB)
