# Supplementary material for: Patent foramen ovale diagnosis in young stroke patients: analysis of recurrence and mortality
Source: J Neurol. 2025 Jun 11;272(7):456. doi: 10.1007/s00415-025-13178-x (PMC12159083; doi:10.1007/s00415-025-13178-x)
Supplement: Supplementary file 1 — Supplementary file1 (DOCX 607 KB) [file 415_2025_13178_MOESM1_ESM.docx]

| **Supplementary table 1. ICD codes of vascular risk factors and main comorbidities.** | | |
| --- | --- | --- |
|  | **ICD-9 codes** | **ICD-10 codes** |
| Patent foramen oval | 745.5 | Q21.1 |
| **Vascular risk factors** | | |
| Hypertension | 401 | I10.xx |
| Diabetes | 250 | E10.xx ,E11.xx, E13.xx |
| Dyslipidemia | 272 | E78.xx |
| Atrial fibrillation | 472.3 | I48.xx |
| Obesity | 278 | E66.xx |
| **Previous Comorbidities** | | |
| Stroke | 433, 434, 435 (and subcodes) | I63.xx except I63.6 |
| Ischemic heart disease | 410,411,412, 413 (and subcodes) | I20.xx, I21.xx,I22.xx, I23.xx, I24.xx, I25.xx |
| Thrombophilia | 289.81 | D68.5.xx, D68.6.xx, D68.8.xx, D68.9.xx |
| Venous thrombosis | 453.4, 453.5, 453.6, 453.7, 453.8 |  |
| Migraine | 346 | G43.xx |

|  | **Supplementary table 2. Main characteristics in younger subjects (≤50 years) and by presence of PFO.** | | | |
| --- | --- | --- | --- | --- |
|  | **Whole cohort** | **Stroke patients with PFO** | **Stroke patients without PFO** |  |
|  | N=5143 | N=436 | N=4707 | *p*-value |
| **Demographics** | | | | |
| Age, years | 45 (39-48) | 45.0 (40-48) | 43 (38-47) | <0.001 |
| Age in decades |  |  |  |  |
| 18-29 | 326 (6.34) | 284 (6.03) | 42 (9.63) | <0.001 |
| 30-39 | 984 (19.1) | 878 (18.7) | 106 (24.3) |  |
| 40-49 | 3257 (63.3) | 3012 (64.0) | 245 (56.2) |  |
| 50y | 576 (11.2) | 533 (11.3) | 43 (9.86) |  |
| Sex, female | 2067 (40.2) | 1881 (40.1) | 179 (41.1) | 0.739 |
| Socioeconomic status |  |  |  | <0.001 |
| <18,000€ | 3905 (79.2) | 293 (68.9) | 3612 (80.2) |  |
| 18,000-100,000€ | 1002 (20.3) | 129 (30.4) | 873 (19.4) |  |
| >100,000€ | 23 (0.5) | 3 (0.7) | 20 (0.4) |  |
| **Vascular risk factors** | | | | |
| Hypertension | 1255 (24.4) | 52 (11.9) | 1203 (25.6) | <0.001 |
| Diabetes | 530 (10.3) | 20 (4.59) | 510 (10.8) | <0.001 |
| Dyslipidemia | 956 (18.6) | 60 (13.8) | 896 (19.0) | 0.008 |
| Atrial fibrillation | 214 (4.2) | 12 (2.75) | 202 (4.29) | 0.157 |
| Obesity | 1192 (23.2) | 77 (17.7) | 1115 (23.7) | 0.005 |
| **Previous comorbidities** | | | | |
| Stroke | 534 (10.4) | 55 (12.6) | 479 (10.2) | 0.130 |
| Ischemic heart disease | 161 (3.1) | 7 (1.61) | 154 (3.27) | 0.077 |
| Thrombophilia | 36 (0.7) | 3 (0.69) | 33 (0.70) | 1.000 |
| Venous thrombosis | 26 (0.5) | 1 (0.23) | 25 (0.53) | 0.721 |
| Migraine | 519 (10.1) | 51 (11.7) | 468 (9.94) | 0.280 |
| **Previous use of antiplatelets and oral anticoagulants drugs** | | | | |
| Antiplatelets | 510 (9.9) | 37 (8.49) | 473 (10.0) | 0.337 |
| Anticoagulant | 128 (2.5) | 8 (1.2) | 120 (2.6) | 0.450 |
| **New use of antiplatelets and oral anticoagulants drugs** | | | | |
| Antiplatelets | 2847 (55.4) | 321 (73.6) | 2526 (53.7) | <0.001 |
| Anticoagulant | 401 (7.8) | 69 (15.8) | 332 (7.1) | <0.001 |

Values represent frequencies (percentages) or medians (interquartile ranges). Characteristics between participants with and without PFO have been obtained with 𝜒^2^ or U Mann-Whitney tests, accordingly.

|  | **Supplementary table 3: Differences between stroke patients without PFO diagnosed before and after the update in clinical guidelines (2018)** | | |
| --- | --- | --- | --- |
|  | **Stroke patients recruited from 2016 to 2017** | **Stroke patients recruited from 2019 to 2021** |  |
|  | N= 5350 | N= 6352 | *p*-value |
| **Demographics** | | | |
| Age, years | 53.0 (47.0-57.0) | 53.0 (47.0;57.0) | 0.039 |
| Age in decades |  |  | 0.004 |
| 18-29 | 104 (1.94) | 189 (2.98) |  |
| 30-39 | 377 (7.05) | 445 (7.01) |  |
| 40-49 | 1265 (23.6) | 1520 (23.9) |  |
| 50-60 | 3604 (67.4) | 4198 (66.1) |  |
| Sex, female | 1805 (33.7) | 2115 (33.3) | 0.628 |
| Socioeconomic status |  |  | 0.017 |
| <18,000€ | 3853 (74.8) | 4757 (77.1) |  |
| 18,000-100,000€ | 1270 (24.7) | 1381 (22.4) |  |
| >100,000€ | 25 (0.5) | 28 (0.5) |  |
| **Vascular risk factors** | | | |
| Hypertension | 2041 (38.1) | 2406 (37.9) | 0.778 |
| Diabetes | 1061 (19.8) | 1161 (18.3) | 0.035 |
| Dyslipidemia | 1771 (33.1) | 1962 (30.9) | 0.035 |
| Atrial fibrillation | 337 (6.30) | 425 (6.69) | 0.413 |
| Obesity | 1373 (25.7) | 1871 (29.5) | <0.001 |
| **Previous comorbidities** | | | |
| Stroke | 805 (15.0) | 709 (11.2) | <0.001 |
| Ischemic heart disease | 386 (7.21) | 357 (5.62) | <0.001 |
| Thrombophilia | 26 (0.49) | 34 (0.54) | 0.809 |
| Venous thrombosis | 25 (0.47) | 38 (0.60) | 0.402 |
| Migraine | 360 (6.73) | 487 (7.67) | 0.056 |

Values represent frequencies (percentages) or medians (interquartile ranges). Characteristics between groups have been obtained with 𝜒^2^ or U Mann-Whitney tests, accordingly.

|  | **Supplementary table 4: Differences between stroke patients with PFO recruited before and after the update in clinical guidelines (2018)** | | |
| --- | --- | --- | --- |
|  | **Stroke patients recruited from 2016 to 2017** | **Stroke patients recruited from 2019 to 2021** |  |
|  | N=219 | N=413 | *p*-value |
| **Demographics** | | | |
| Age, years | 48.0 (42.0-55.0) | 49.0 (42.0-55.0) | 0.932 |
| Age in decades |  |  | 0.946 |
| 18-29 | 11 (5.02%) | 25 (6.05%) |  |
| 30-39 | 30 (13.7%) | 56 (13.6%) |  |
| 40-49 | 71 (32.4%) | 137 (33.2%) |  |
| 50-60 | 107 (48.9%) | 195 (47.2%) |  |
| Sex, female | 94 (42.9%) | 144 (34.9%) | 0.057 |
| Socioeconomic status |  |  | 0.811 |
| <18,000€ | 140 (66.0%) | 269 (66.6%) |  |
| 18,000-100,000€ | 72 (34.0%) | 133 (32.9%) |  |
| >100,000€ | 0 (0.0%) | 2 (0.5%) |  |
| **Vascular risk factors** | | | |
| Hypertension | 36 (16.4%) | 81 (19.6%) | 0.384 |
| Diabetes | 23 (10.5%) | 27 (6.54%) | 0.109 |
| Dyslipidemia | 48 (21.9%) | 93 (22.5%) | 0.943 |
| Atrial fibrillation | 8 (3.65%) | 17 (4.12%) | 0.944 |
| Obesity | 44 (20.1%) | 90 (21.8%) | 0.693 |
| **Previous comorbidities** | | | |
| Stroke | 36 (16.4%) | 46 (11.1%) | 0.078 |
| Ischemic heart disease | 9 (4.11%) | 11 (2.66%) | 0.454 |
| Thrombophilia | 2 (0.91%) | 1 (0.24%) | 0.277 |
| Venous thrombosis | 1 (0.46%) | 2 (0.48%) | 1.000 |
| Migraine | 23 (10.5%) | 43 (10.4%) | 1.000 |

Values represent frequencies (percentages) or medians (interquartile ranges). Characteristics between groups have been obtained with 𝜒^2^ or U Mann-Whitney tests, accordingly.

| **Supplementary table 5. Effect of time on PFO diagnosis.** | | |
| --- | --- | --- |
| **Variable** | **OR (95% CI)** | ***p*-value** |
| **Dichotomic time** | | |
| Time, 2016-2017 vs 2019-2021 | 1.59 (1.35-1.89) | <0.001 |
| Age, year increase | 0.95 (0.95-0.96) | <0.001 |
| **Discrete time** | | |
| Time, year increase | 1.11 (1.07-1.16) | <0.001 |
| Age, year increase | 0.95 (0.94-0.96) | <0.001 |
| **Ordinal time** | | |
| Time, 2017vs2016 | 0.98 (0.74-1.28) | 0.870 |
| Time, 2018vs2017 | 1.42 (1.08-1.87) | 0.012 |
| Time, 2019vs2018 | 1.11 (0.86-1.44) | 0.424 |
| Time, 2020vs2019 | 1.07 (0.83-1.37) | 0.591 |
| Time, 2021vs2020 | 0.93 (0.73-1.19) | 0.583 |
| Age, year increase | 0.95 (0.95-0.96) | <0.001 |

Logistic regression models showing the association between time —either as a dichotomic, continuous or ordinal variable— and PFO diagnosis (models are adjusted for age). Values represent Odds ratios (OR), 95 % confidence intervals (CI) and *p*-values.

| **Supplementary table 6. Increase in the detection of PFO between the periods before and after the updated in clinical guidelines (2018).** | | |  |
| --- | --- | --- | --- |
|  | **2016-2017** | **2019-2021** | ***p*-value** |
| Whole sample | 219/5350 (4.1) | 413/6352 (6.5) |  |
| **Stratified by age** | | |  |
| 18-29 | 11/104 (10.6) | 25/189 (13.2) | 0.416 |
| 30-39 | 30/377 (7.9) | 56/445 (12.6) |  |
| 40-49 | 71/1265 (5.6) | 137/1520 (9.0) |  |
| 50-60 | 107/3604 (3.0) | 195/4198 (4.6) |  |
| **Stratified by sex** | | |  |
| Female | 94/1805 (5.2) | 144/2115 (6.8) | 0.070 |
| Male | 125/3545 (3.5) | 269/4237 (6.3) |  |
| **Stratified by region** | | |  |
| Alt Pirineu i Aran | 2/52 (3.8) | 3/51 (5.9) | 0.837 |
| Barcelona | 41/861 (4.7) | 108/1208 (8.9) |  |
| Barcelona - North | 51/1500 (3.4) | 86/1652 (5.2) |  |
| Barcelona - South | 45/857 (5.3) | 83/991 (8.3) |  |
| Camp de Tarragona | 11/354 (3.1) | 28/443 (6.3) |  |
| Central Catalonia | 14/315 (4.4) | 18/315 (5.7) |  |
| Girona | 22/594 (3.7) | 47/734 (6.4) |  |
| Lleida | 11/227 (4.8) | 16/314 (5.1) |  |
| Penedès | 8/298 (2.7) | 9/400 (2.25) |  |
| Terres de l'Ebre | 7/132 (5.3) | 13/166 (7.8) |  |

Values indicate the proportion of patients with a PFO within a specific stratum. *P*-values represent the interaction term between age, sex or region with time on the proportion of stroke cases with PFO.

| **Supplementary table 7: Moderation of age, sex and region on the relationship between time and PFO diagnosis** | |
| --- | --- |
| **Variable** | ***p*-value** |
| **Discrete time** | |
| Age*Time | 0.218 |
| Sex*Time | 0.022 |
| Region*Time | 0.892 |
| **Ordinal time** | |
| Age*Time | 0.739 |
| Sex*Time | 0.106 |
| Region*Time | - |

Values indicate the *p*-value of the interaction term between age, sex or region with time on the proportion of stroke cases with PFO. Binomial logistic regression models were constructed with the presence of PFO as the dependent variable, adjusting for time and age. Three different models were built to test the moderation effects of age, sex, and region on the relationship between time and the increase in PFO diagnoses.

|  | **Supplementary table 8: Differences between stroke patients included and excluded patients in the stroke recurrence study** | | |
| --- | --- | --- | --- |
|  | **Excluded** | **Included** |  |
|  | N=4600 | N=9180 | *p*-value |
| **Demographics** | | | |
| Age, years | 52.0 (44-57) | 54 (48-57) | <0.001 |
| Age in decades |  |  | <0.001 |
| 18-29 | 189 (4.1) | 137 (1.5) |  |
| 30-39 | 480 (10.4) | 504 (5.5) |  |
| 40-49 | 1170 (25.4) | 2087 (22.7) |  |
| 50-60 | 2761 (60.0) | 6452 (70.3) |  |
| Sex, female | 1767 (38.4) | 6350 (30.8) | <0.001 |
| Socioeconomic status |  |  | 0.031 |
| <18,000€ | 3406 (77.8) | 6781 (75.8) |  |
| 18,000-100,000€ | 948 (21.7) | 2119 (23.7) |  |
| >100,000€ | 24 (0.6) | 45 (0.5) |  |
| **Vascular risk factors** | | | |
| Hypertension | 1628 (35.4) | 3640 (39.7) | <0.001 |
| Diabetes | 807 (17.5) | 1808 (19.7) | 0.003 |
| Dyslipidemia | 1337 (29.1) | 3081 (33.6) | <0.001 |
| Atrial fibrillation | 265 (5.8) | 662 (7.2) | <0.001 |
| Obesity | 1231 (26.8) | 2622 (28.6) | 0.028 |
| **Previous comorbidities** | | | |
| Stroke | 1789 (38.9) | 0 (0) | - |
| Ischemic heart disease | 245 (5.3) | 635 (6.9) | 0.454 |
| Thrombophilia | 25 (0.5) | 44 (0.5) | 0.707 |
| Venous thrombosis | 23 (0.5) | 58 (0.6) | 0.403 |
| Migraine | 422 (9.2) | 549 (6.0) | <0.001 |

Values represent frequencies (percentages) or medians (interquartile ranges). Characteristics between groups have been obtained with 𝜒^2^ or U Mann-Whitney tests, accordingly.

| **Supplementary table 9. Association between demographic and vascular risk factors with stroke recurrence in the whole sample (N=9,136)** | | | |
| --- | --- | --- | --- |
| **Variable** | **Group** | **1-(Survival-Rate)** | ***p*-value** |
| Age ≤ 50 years | ≤ 50 | 11.7 (10.4;13.1) | 0.265 |
|  | > 50 | 12.3 (11.4;13.3) |  |
| Age in decades | 18-29 | 21.1 (10.5;31.7) | 0.163 |
|  | 30-39 | 13.0 (9.4;16.6) |  |
|  | 40-49 | 11.2 (9.6;12.9) |  |
|  | 50-60 | 12.2 (11.3;13.1) |  |
| Sex | Female | 10.9 (9.5;12.3) | 0.036 |
|  | Male | 12.6 (11.7;13.6) |  |
| Socioeconomic status | <18,000€ | 12.9 (12.0;13.9) | <0.001 |
|  | ≥18,000€ | 9.2 (7.7;10.6) |  |
| PFO diagnosis | No | 12.1 (11.3;12.9) | 0.830 |
|  | Yes | 12.9 (9.7;16.2) |  |
| Hypertension | No | 11.6 (10.6;12.5) | 0.265 |
|  | Yes | 12.9 (11.6;14.2) |  |
| Diabetes | No | 11.1 (10.3;12) | <0.001 |
|  | Yes | 16.0 (14.0;18.1) |  |
| Dyslipidemia | No | 11.4 (10.5;12.4) | 0.063 |
|  | Yes | 13.4 (12.0;14.8) |  |
| Atrial fibrillation | No | 12.1 (11.3;13.0) | 0.990 |
|  | Yes | 11.8 (8.9;14.7) |  |
| Obesity | No | 11.9 (11.0;12.9) | 0.467 |
|  | Yes | 12.5 (11.0;14) |  |
| Previous ischemic cardiopathy | No | 11.8 (11.0;12.6) | <0.001 |
|  | Yes | 16.6 (13.4;19.9) |  |
| Previous thrombophilia | No | 12.2 (11.4;12.9) | 0.239 |
|  | Yes | 4.5 (0.0;10.7) |  |
| Previous venous thrombosis | No | 12.1 (11.3;12.9) | 0.325 |
|  | Yes | 16.5 (5.8;27.2) |  |
| Migraine | No | 12.2 (11.4;13.1) | 0.150 |
|  | Yes | 10.1 (7.0;13.1) |  |

Values represent the rate of events (1-survival function) and 95% confidence intervals. The *p*-values have been obtained via log-Rank tests.

| **Supplementary table 10. All-cause mortality in the sample (N=9,070).** | | | |
| --- | --- | --- | --- |
| **Year** | **Survival rate (95% CI)** | **N at risk** | **N events** |
| Whole Sample | | | |
| 1 | 95.4 (95.0-95.8) | 7151 | 396 |
| 2 | 93.3 (92.8-93.9) | 5298 | 137 |
| 3 | 91.2 (90.6-91.9) | 3626 | 100 |
| 4 | 88.7 (87.9-89.6) | 1697 | 72 |
| By Age Groups | | | |
| *18-29* |  |  |  |
| 1 | 98.0 (95.3-100.0) | 84 | 2 |
| 2 | 98.0 (95.3-100.0) | 59 | 0 |
| 3 | 98.0 (95.3-100.0) | 43 | 0 |
| 4 | 95.6 (90.4-100.0) | 23 | 1 |
| *30-39* |  |  |  |
| 1 | 96.6 (94.9-98.3) | 371 | 15 |
| 2 | 95.9 (94.0-97.9) | 277 | 2 |
| 3 | 93.9 (91.4-96.5) | 175 | 5 |
| 4 | 93.2 (90.4-96.2) | 77 | 1 |
| *40-49* |  |  |  |
| 1 | 97.1 (96.4-97.9) | 1555 | 55 |
| 2 | 95.6 (94.7-96.6) | 1132 | 20 |
| 3 | 94.4 (93.3-95.6) | 770 | 12 |
| 4 | 93.0 (91.4-94.5) | 373 | 8 |
| *50-60* |  |  |  |
| 1 | 94.7 (94.2-95.3) | 5141 | 324 |
| 2 | 92.3 (91.7-93.0) | 3830 | 115 |
| 3 | 90.0 (89.2-90.9) | 2638 | 83 |
| 4 | 87.1 (86.0-88.2) | 1224 | 62 |

Values represent the survival function obtained via the Kaplan-Meier estimator. The event of interest was all-cause mortality, although excluding the index stroke-related mortality.

**Supplementary figure 1.** Period covered by each database. Bolded years (2016 to 2021) indicate the study period.

**
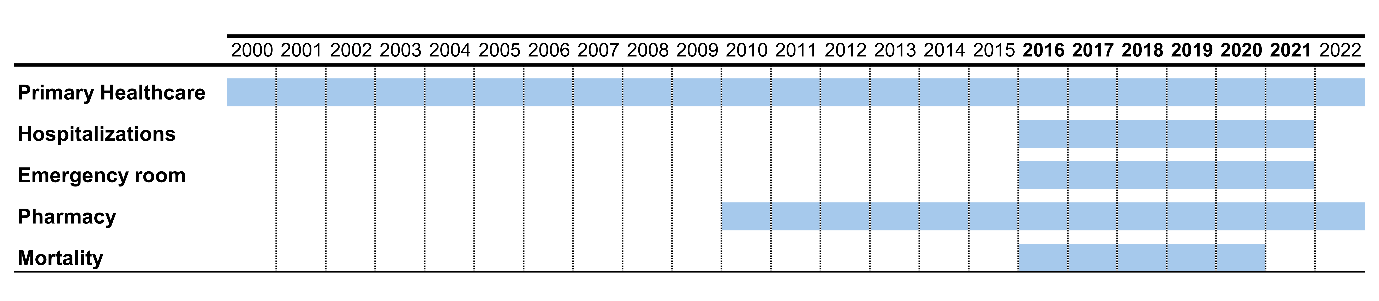
**

**Supplementary figure 2.** Study flow-chart.

**
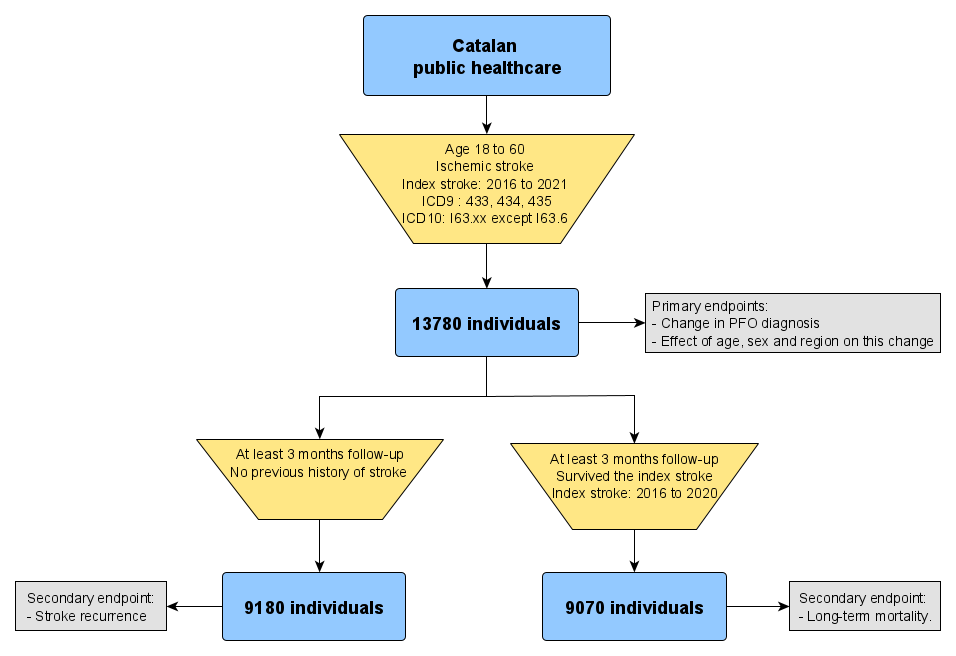
**

**Supplementary figure 3.** Incidence of stroke at younger ages. Panel A depicts the age-specific incidence rates per 100,000 inhabitants. Panel B stratifies the incidence rates by age groups.

**
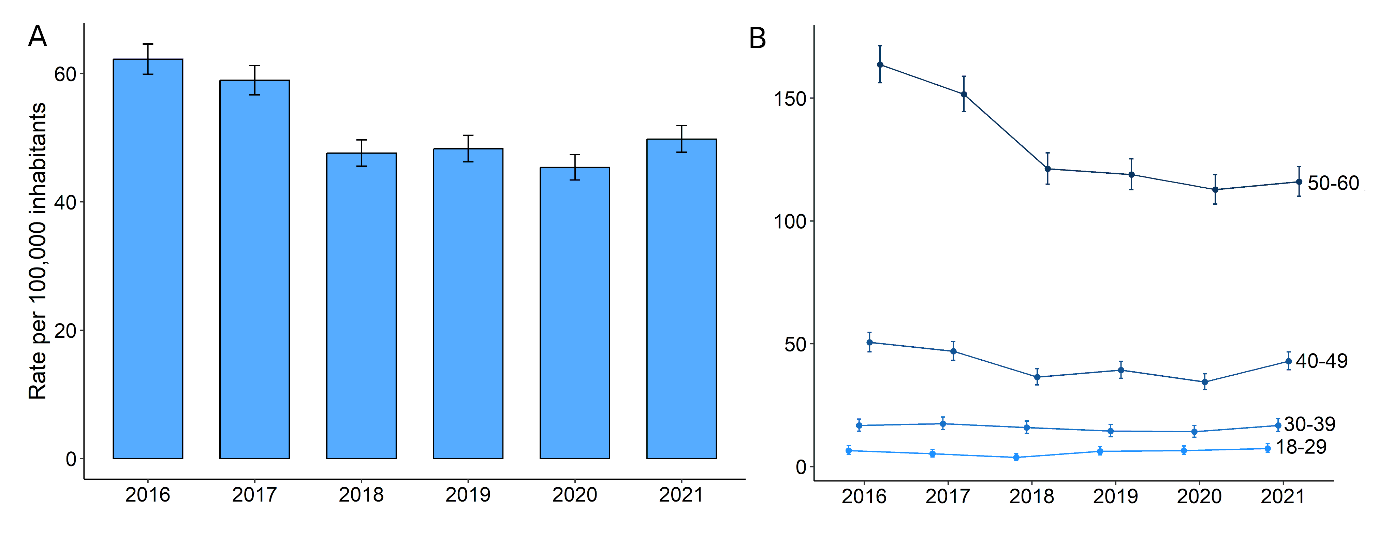
**

**Supplementary figure 4.** Time from stroke to PFO diagnosis.


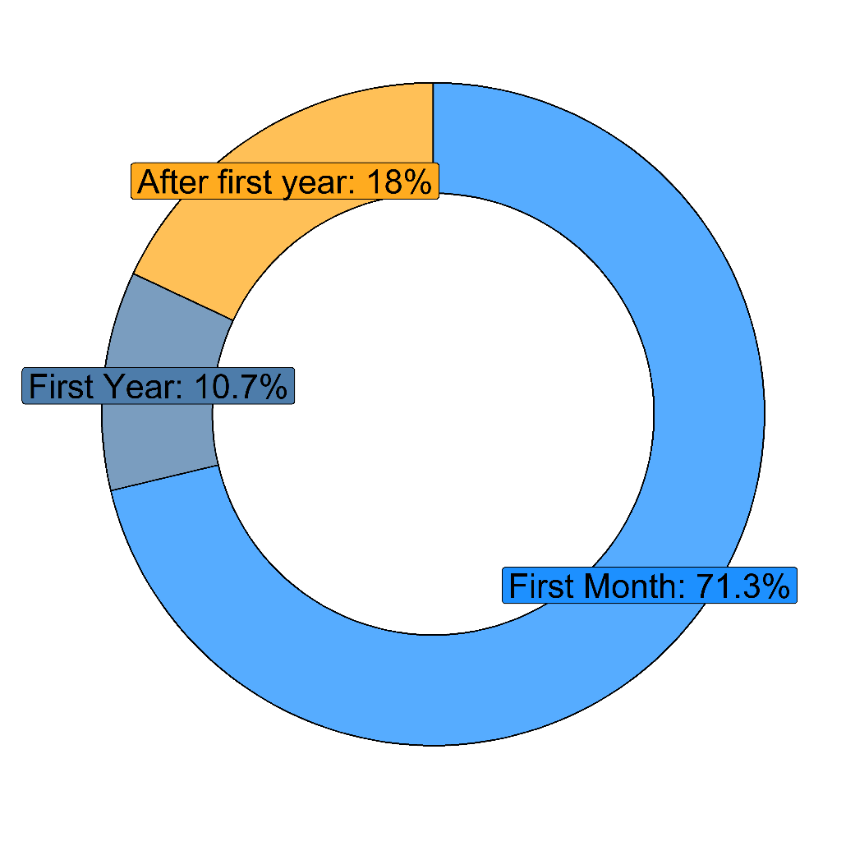


**Supplementary figure 5.** Effect of territory in the increase of PFO diagnosis. Panel A contrasts the incidence of strokes associated with PFO before (2016-2017, blue) and after (2019-2021, orange) the update of clinical guidelines. In Panel B, we depict the linear correlation between time and the frequency of PFO diagnoses across Catalonia's map.

**
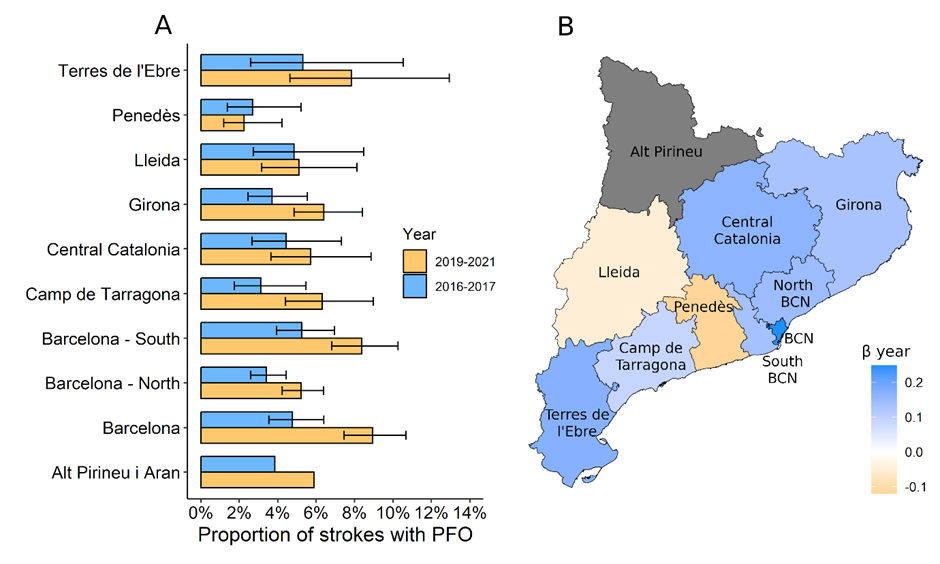
**

**Supplementary figure 6**. Kaplan-Meier curve depicting the 5-year incidence of stroke recurrences in the sample. Values represent the accumulated number of events and patients at risk for each year.
